# Orthodontic Root Resorption in Spontaneously Hypertensive Rats: Modulation by β2-Adrenergic Receptor Blockade

**DOI:** 10.3390/jcm15187271

**Published:** 2026-09-18

**Authors:** Tomoma Yoshida, Takuma Sato, Mifumi Takahashi, Shota Ichikawa, Masako Tabuchi, Ken Miyazawa

**Affiliations:** Department of Orthodontics, School of Dentistry, Aichi Gakuin University, 2-11 Suemori-dori, Chikusa-ku, Nagoya 464-8651, Japan; tomoma42954@gmail.com (T.Y.); tmifumi@dpc.agu.ac.jp (M.T.); soccerbaka.77@icloud.com (S.I.); machako@dpc.agu.ac.jp (M.T.); miyaken@dpc.agu.ac.jp (K.M.)

**Keywords:** hypertension, orthodontically induced inflammatory root resorption, sympathetic nervous system, β2-adrenergic receptor, butoxamine, odontoclast, cementocyte

## Abstract

**Background/Objectives**: Hypertension is associated with abnormal bone metabolism and enhanced bone resorption, and altered sympathetic/adrenergic signaling has been proposed as one contributing mechanism. Orthodontically induced inflammatory root resorption (OIRR) involves periodontal ligament inflammation, odontoclast induction, and the resorption of cementum and dentin. This study investigated whether the SHR phenotype is associated with greater OIRR and whether the pharmacological blockade of β2-adrenergic receptor (β2-AR) signaling with butoxamine (BTX) modulates these changes. **Methods**: A 50 gf Ni-Ti closed-coil spring was placed between the maxillary incisors and left first molar in SHRs and Wistar–Kyoto (WKY) rats for 21 days, beginning 7 days after BTX administration. Animals were assigned to WKY(control), WKY(BTX), SHR(control), and SHR(BTX) groups. The tooth-movement distance, root length, and root volume were assessed via micro-computed tomography. The odontoclast number and surface, TNF-α, IL-1β, and β2-AR immunoreactivity, cementocyte density, empty lacunae ratio, and TUNEL-positive cells within cementocyte lacunae were evaluated histologically. **Results**: Compared with WKY(control), SHR(control) showed greater tooth movement, a reduced root length and volume, an increased odontoclast number and surface, greater TNF-α- and IL-1β-positive areas, a lower cementocyte density, a higher empty lacunae ratio, and more TUNEL-positive cells within cementocyte lacunae. These root resorption- and inflammation-related changes were significantly attenuated in SHR(BTX) compared with SHR(control). **Conclusions**: The SHR phenotype was associated with increased OIRR, and the attenuation observed following β2-AR blockade supports a potential contribution of adrenergic signaling to this response.

## 1. Introduction

Hypertension is a highly prevalent chronic disease worldwide and has been shown to affect not only the cardiovascular system but also bone metabolism [1,2]. Recent epidemiological studies and meta-analyses have revealed reduced bone mineral density and an increased risk of fracture in patients with hypertension, suggesting that hypertension may be associated with systemic abnormalities in bone metabolism [3,4]. Altered sympathetic/adrenergic signaling has been proposed as one possible mechanism contributing to the abnormalities in bone metabolism associated with hypertension. Sympathetic neurotransmitters are known to influence bone remodeling through the β2-adrenergic receptors (β2-ARs) expressed on osteoblasts and osteoclasts [5,6].

Interestingly, studies using spontaneously hypertensive rats (SHRs) have shown that the administration of butoxamine (BTX), a selective β2-AR antagonist, markedly suppresses alveolar bone loss and reduces tooth movement [7,8]. Kondo et al. reported that experimental tooth movement in mice increases sympathetic nerve fibers (tyrosine hydroxylase [TH]-positive nerve fibers) within the periodontal ligament tissue and enhances osteoclast activity. In addition, sensory nerve injury or sympathectomy suppresses both tooth movement and osteoclast activity, suggesting that a neural loop involving the sensory nervous system, central nervous system, and sympathetic nervous system contributes to the regulation of tooth movement [9]. These findings suggest that altered sympathetic/adrenergic signaling in the SHR model may contribute to enhanced alveolar bone resorption [10,11]. However, these studies focused primarily on alveolar bone resorption and tooth movement and did not sufficiently investigate the association of the SHR phenotype and β2-adrenergic signaling with orthodontic root resorption.

Orthodontically induced inflammatory root resorption (OIRR) is one of the major adverse effects associated with orthodontic treatment and is characterized by the resorption of cementum and dentin. The development of OIRR involves impaired blood flow caused by periodontal ligament compression, hyalinization, inflammatory cytokine responses, and odontoclast induction. In particular, excessive compressive stimuli that increase intraperiodontal pressure beyond capillary pressure have been suggested to promote odontoclast activation and root resorption [12,13,14,15]. However, systemic arterial hypertension and local compressive pressure within the periodontal ligament represent distinct physiological phenomena, and a direct relationship between them has not been established. In addition, OIRR shares osteoclast/odontoclast-dependent mechanisms with alveolar bone resorption, and root resorption and alveolar bone resorption are thought to involve common biological mechanisms [12,16]. However, it remains unclear whether the SHR phenotype is associated with greater OIRR under orthodontic force and whether the blockade of β2-AR signaling can attenuate OIRR-related changes.

In this study, we used an experimental tooth-movement model with continuous orthodontic force to investigate OIRR in SHRs and the effects of β2-AR blockade. Based on previous findings showing that β2-AR blockade modulates tooth movement and alveolar bone resorption in SHRs [8,17], we hypothesized that SHRs would exhibit greater OIRR than WKY rats and that β2-AR blockade would attenuate this response. We therefore evaluated root morphology, odontoclast activity, inflammatory responses, and cementocyte-related changes.

## 2. Materials and Methods

### 2.1. Animals and Reagents

Seven-week-old male SHR/Izm and Wistar Kyoto (WKY)/Izm rats (Japan SLC, Inc., Shizuoka, Japan) were housed, 3 or 4 per cage, under automatically controlled environmental conditions (temperature, 23 ± 1 °C; humidity, 50 ± 10%) with a 12 h light/dark cycle and were allowed free access to tap water and standard laboratory chow. All rats were acclimatized to the housing conditions for 1 week. Male rats were used to maintain consistency with our previous studies using the SHR orthodontic tooth-movement model and to facilitate a comparison with our earlier findings. Animal care and all experimental procedures were approved by the Animal Experimentation Committee of the School of Dentistry, Aichi Gakuin University (approval no. AGUD533; approved on 1 April 2025) and conducted in accordance with the institutional guidelines for animal experimentation. Throughout the experimental period, all rats were monitored daily for general health, body weight, respiration, appearance, coat condition, and activity. Humane euthanasia was planned if severe distress, marked hypoactivity, difficulty in feeding or drinking, or a body weight loss of more than 20% was observed. One animal in the WKY(control) group died before completion of the experiment. No other unexpected adverse events were observed during the experimental period. BTX was purchased from Sigma-Aldrich (St. Louis, MO, USA). Within each strain, rats were randomly allocated to the control or BTX groups by a blinded staff member not involved in the study using a computer-generated random number table. Rats in the SHR(BTX) and WKY(BTX) groups received BTX orally via gastric gavage at a dose of 1.0 mg/kg once daily for 4 weeks, while rats in the SHR(control) and WKY(control) groups received an equivalent volume of saline. The BTX dose was selected based on our previous studies using the SHR model, including the dose-dependent effects of BTX on bone metabolism and its use in the same SHR orthodontic tooth-movement model [7,8]. At the end of the experiment, the maxillae were excised. An a priori sample-size calculation was performed using G*Power software version 3.1.9.6 (Heinrich-Heine-Universität Düsseldorf, Düsseldorf, Germany) based on an overall omnibus F-test among the four experimental groups. The experimental design was informed by our previous study using a similar orthodontic tooth-movement model in WKY and SHR rats with or without butoxamine treatment [8]. Based on the substantial between-group differences reported in that study, a large standardized effect size (Cohen’s f = 0.5) was assumed. With an α level of 0.05 and a statistical power of 0.80, the required total sample size was calculated to be 48 animals (12 animals per group). Potential animal loss or orthodontic appliance failure was considered during study planning; however, because the extent of such losses could not be reliably predicted in advance, no additional inflation of the calculated sample size was applied. During the experimental period, one animal in the WKY(control) group died, and orthodontic appliance loss occurred in four WKY(control), two WKY(BTX), and two SHR(BTX) animals. Consequently, the final sample sizes for the main experimental analyses were 7, 10, 12, and 10 animals in the WKY(control), WKY(BTX), SHR(control), and SHR(BTX) groups, respectively. For the histological and immunohistochemical analyses, specimens were randomly selected from the animals available after completion of the main experiment and allocated to the planned analyses. Consequently, the number of animals analyzed differed among individual histological and immunohistochemical outcomes. No single primary outcome was prespecified before the experiment; accordingly, the a priori sample-size calculation was based on an overall comparison among the four experimental groups rather than on a specific outcome. The study was designed to evaluate multiple complementary morphological, histological, and inflammatory outcomes related to OIRR.

### 2.2. Experimental Tooth Movement

**Figure 1:**
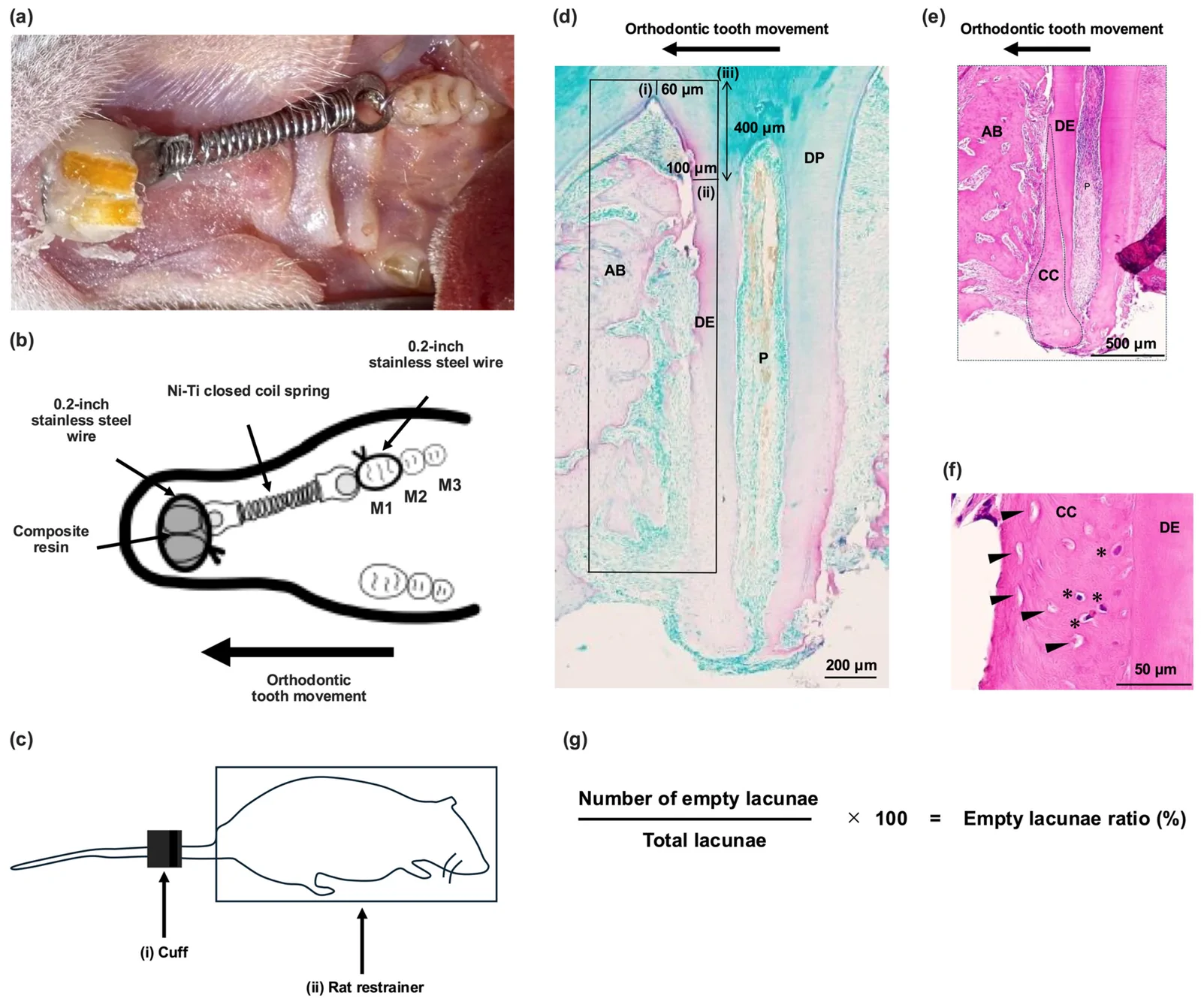
Schematic illustration of the experimental procedures. (**a**,**b**) Intraoral photograph of a rat after placement of the tooth movement appliance (**a**) and schematic occlusal view (**b**). (**c**) Method for measuring blood pressure in rats (i, cuff; ii, rat restrainer). (**d**) Measurement area for TRAP staining. A rectangular region measuring 2000 × 500 μm^2^ and oriented parallel to the long axis of the distopalatal root of the maxillary first molar was defined as the measurement area for TRAP staining. The upper border of the rectangle was positioned 60 μm coronal to the highest point of the furcation (i). The distal border was positioned 100 μm into the dentin (ii), and its starting point was positioned 400 μm below the upper border of the rectangle (iii). (**e**) Hematoxylin and eosin-stained image showing the measurement area for cellular cementum on the compression side of the distopalatal root of the maxillary left first molar (dashed outline = cellular cementum). (**f**) Hematoxylin and eosin-stained image of cellular cementum. Asterisks indicate cementocytes, and black arrowheads indicate empty lacunae. (**g**) Equation for calculating the empty lacunae ratio. AB, alveolar bone; CC, cellular cementum; DE, dentin; DP, distopalatal root; M1, maxillary first molar; M2, maxillary second molar; M3, maxillary third molar; P, pulp; TRAP, tartrate-resistant acid phosphatase.

Experimental tooth movement was initiated 1 week after the start of drug administration. The rats were anesthetized via the intraperitoneal administration of a mixture of medetomidine hydrochloride (Meiji Seika Pharma Co., Ltd., Tokyo, Japan), midazolam (Astellas Pharma Inc., Tokyo, Japan), and butorphanol tartrate (Meiji Seika Pharma Co., Ltd., Tokyo, Japan). In accordance with the methods described by Dunn et al. [18] and Sato et al. [8], a Ni-Ti closed-coil spring (Sentalloy^®^, Tomy, Tokyo, Japan) was attached between the maxillary incisors and the maxillary left first molar using a 0.020-inch ligature wire to move the left first molar mesially for 3 weeks. The force delivered by each spring was verified at appliance placement using a force gauge to ensure a force of 50 gf. Appliance integrity was checked throughout the 21-day experimental period. However, force decay was not quantitatively measured during this period. Composite resin (TRANSBOND PLUS; 3M Unitek, Monrovia, CA, USA) was applied over the ligature wire on the incisors to prevent slippage and pulpal irritation from exposed dentin (Figure 1a,b).

### 2.3. Analysis of Tooth Movement Distance and Root Morphology Using Micro-Computed Tomography

Three weeks after the initiation of experimental tooth movement, the maxillae were excised and subjected to three-dimensional (3D) structural analysis using micro-computed tomography (μCT; Rigaku, Tokyo, Japan). The scanning parameters were as follows: tube voltage, 90 kV; tube current, 150 μA; scanning time, 2 min; and voxel size, 20 × 20 × 20 μm. The distance of tooth movement was evaluated by measuring the narrowest distance between the maxillary left first and second molars. Root length was measured on reconstructed 3D images using TRI/3D-BON software version R.12.00.03.9-H-64 (RATOC System Engineering, Tokyo, Japan). The furcation and root apex of the distopalatal root were identified as anatomical landmarks, and the three-dimensional linear distance between these two points along the long axis of the root was measured as the root length. In addition, the volume of the distopalatal root of the maxillary first molar was measured by defining the region from the furcation to the root apex as the region of interest (ROI), segmenting the root hard tissue from the surrounding tissues on reconstructed 3D images, and measuring the segmented root volume using TRI/3D-BON software. The same segmentation threshold was applied consistently to all specimens. All μCT measurements and segmentation procedures were performed by an assessor blinded to strain and treatment allocation.

### 2.4. Blood Pressure Measurement

Systolic blood pressure was measured using the tail-cuff method with a noninvasive automatic blood pressure monitoring system for rats and mice (BP-98A-L; Natsume, Tokyo, Japan). Before data collection, the rats were acclimatized to the restraint and tail-cuff measurement procedure. On each measurement day, the warming chamber was set to 39 °C, and after 5 min of warming, the cuff was placed between the proximal and middle portions of the tail while the rat was in a stable condition. Measurements were started once the pulse had stabilized, and the mean of three measurements was used for analysis. Blood pressure was measured at the beginning and end of the 21-day experimental tooth-movement period. BTX was administered immediately after the blood pressure measurement (Figure 1c). Systolic blood pressure could not be obtained from one animal in the WKY(BTX) group because stable tail-cuff measurements could not be achieved during the measurement session; therefore, the blood pressure analysis included 9 animals in this group.

### 2.5. Histopathological Examination

The harvested maxillae were fixed in 10% neutral-buffered formalin and decalcified in 10% EDTA (pH 7.2) at 4 °C for 4 weeks. The specimens were embedded in paraffin and cut into serial mesiodistal sections of 5 μm in thickness. The observation plane was selected so that all roots in the molar region could be examined. For each animal, three sections representing comparable anatomical levels of the distopalatal root were selected from the serial sections and analyzed. Measurements obtained from the three sections were averaged to generate a single animal-level value for statistical analysis. Histological assessments were performed by assessors blinded to the strain and treatment allocation.

To measure the number of odontoclasts per root surface (Oc.N/BS) and odontoclast surface per root surface (Oc.S/BS), tartrate-resistant acid phosphatase (TRAP) staining was performed using a TRAP staining kit (FUJIFILM Wako Pure Chemical Corporation, Osaka, Japan). Based on the method of Mavragani et al. [16], a rectangular ROI measuring 2000 × 500 μm^2^ was defined on the mesial side of the distopalatal root of the maxillary first molar in the TRAP-stained sections. Within the ROI, TRAP-positive multinucleated cells in contact with the root surface or located within resorption lacunae were defined as odontoclasts. The number of odontoclasts and the proportion of the root surface covered by odontoclasts were measured using ImageJ (ver. 1.54g, National Institutes of Health, Bethesda, MD, USA) (Figure 1d).

Hematoxylin and eosin staining was performed to determine the number of cementocytes per unit area of cellular cementum and the empty lacunae ratio on the compression side of the distopalatal root of the maxillary first molar. For each selected section, the entire cellular cementum region delineated in Figure 1e was quantified, rather than selected microscopic fields. The empty lacunae ratio was calculated as the number of empty lacunae divided by the sum of the numbers of empty lacunae and cementocytes within the cellular cementum and expressed as a percentage (Figure 1e–g).

Next, TUNEL (terminal deoxynucleotidyl transferase dUTP nick end labeling) staining was performed using a TUNEL Assay Kit–HRP-DAB (ab66110; Abcam, Cambridge, UK) to detect DNA fragmentation. TUNEL-positive cells located within cementocyte lacunae in the cellular cementum on the mesial side of the distopalatal root of the maxillary first molar were counted. The TUNEL-positive cell ratio was calculated as the number of TUNEL-positive cells divided by the sum of TUNEL-positive cells and cementocytes and expressed as a percentage.

Immunohistochemical staining for tumor necrosis factor (TNF)-α was performed using Histofine Simple Stain Rat MAX-PO and Histofine Simple Stain DAB solution (Nichirei Bioscience Inc., Tokyo, Japan), along with an anti-TNF-α antibody (ab6671; 1:250; Abcam Inc., Waltham, MA, USA). The TNF-α-positive area within the periodontal ligament space between the first molar and the alveolar bone was quantitatively measured and expressed as a percentage of the total periodontal ligament area. Immunohistochemical staining for IL-1β and β2-AR was performed using Histofine Simple Stain Rat MAX-PO and Histofine Simple Stain DAB solution (Nichirei Bioscience Inc., Tokyo, Japan), along with an anti-IL-1β antibody (ab205924; 1:500; Abcam Inc.) and an anti-β2-AR antibody (ab182136; 1:100; Abcam Inc.). The IL-1β-positive area and β2-AR-positive area within the periodontal ligament were quantitatively measured and expressed as percentages of the total periodontal ligament area. Digital image analysis was performed using ImageJ. The DAB staining component was separated from the counterstain using the Colour Deconvolution 2 plugin (v2.1) with the H DAB vector. The periodontal ligament was defined as the region of interest, and the DAB-positive area was identified via threshold-based segmentation and automatically quantified. The same thresholding procedure was applied consistently to all specimens. Image analysis was performed independently by multiple assessors blinded to strain and treatment allocation, and the mean of their measurements was used for statistical analysis. The formal intra- or inter-examiner repeatability of the image-analysis measurements was not assessed.

### 2.6. Statistical Processing

The experimental data are presented as the mean ± standard deviation. For continuous outcomes measured at a single time point, two-way analysis of variance was performed with the strain (WKY/SHR) and treatment (control/BTX) as factors. The main effects of strain and treatment and the strain × treatment interaction were evaluated, followed by Šídák-adjusted multiple comparisons where appropriate. The normality of model residuals was assessed using the Shapiro–Wilk test, and homogeneity of variance was assessed using Levene’s test. When significant departures from these assumptions were detected, sensitivity analyses were performed using heteroscedasticity-consistent HC3 robust standard errors to assess the robustness of the statistical inferences. These sensitivity analyses did not materially alter the principal conclusions. Body weight and systolic blood pressure data were analyzed using linear mixed-effects models, with strain, treatment, time, and their interactions as fixed effects and the animal as a random effect to account for repeated measurements within the same animal. Comparisons between two independent groups in the supplementary analyses were performed using an unpaired Student’s *t*-test. All statistical analyses were performed using IBM SPSS Statistics Ver. 30 (IBM, Armonk, NY, USA). A *p*-value < 0.05 was considered statistically significant.

## 3. Results

### 3.1. Changes in Body Weight

**Figure 2:**
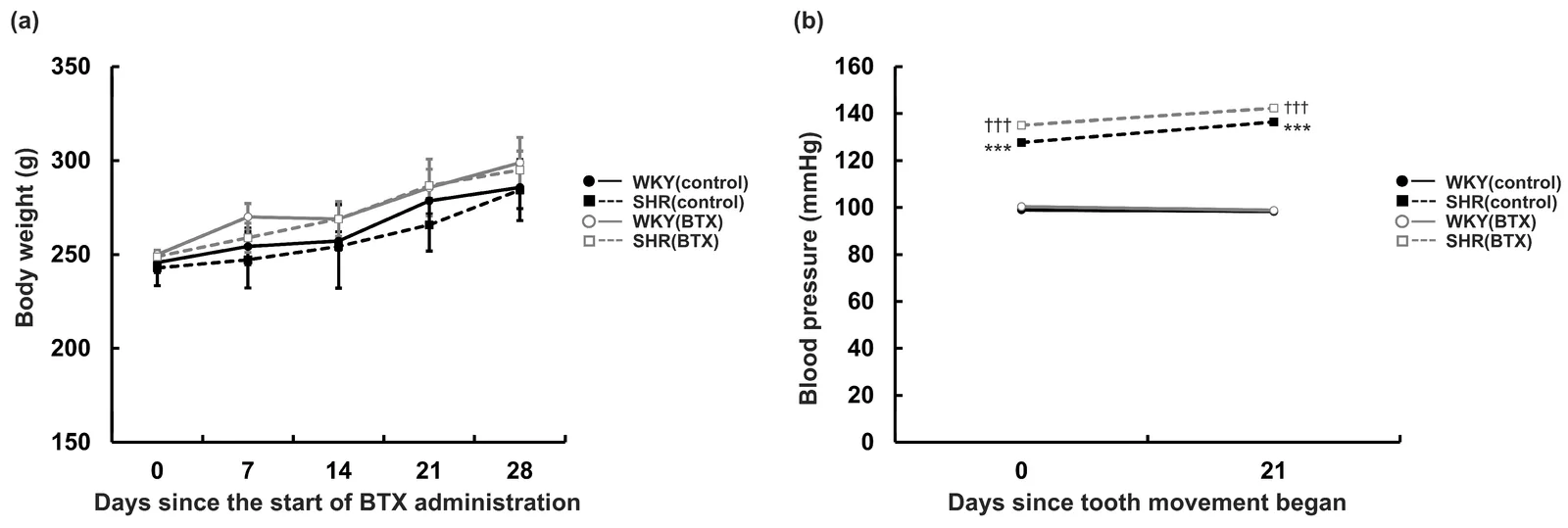
Comparison of body weight and blood pressure. (**a**) Changes in body weight from the start to end of BTX administration (WKY(control), *n* = 7; WKY(BTX), *n* = 10; SHR(control), *n* = 12; SHR(BTX), *n* = 10). (**b**) Comparison of blood pressure at the start and end of tooth movement (WKY(control), *n* = 7; WKY(BTX), *n* = 9; SHR(control), *n* = 12; SHR(BTX), *n* = 10). *** *p* < 0.001 vs. WKY(control), ††† *p* < 0.001 vs. WKY(BTX). BTX, butoxamine; SHRs, spontaneously hypertensive rats; WKY, Wistar–Kyoto rats.

The linear mixed-effects model revealed significant main effects of time (F(4, 88.28) = 100.24, *p* < 0.001) and treatment (F(1, 36.83) = 5.72, *p* = 0.022), whereas the main effect of strain (F(1, 36.83) = 2.02, *p* = 0.164) was not significant. The strain × treatment (F(1, 36.83) = 0.04, *p* = 0.836), strain × time (F(4, 88.28) = 0.25, *p* = 0.906), treatment × time (F(4, 88.28) = 0.44, *p* = 0.783), and strain × treatment × time interactions (F(4, 88.28) = 0.26, *p* = 0.902) were not significant. Šídák-adjusted comparisons showed no significant difference between the control and BTX groups at Day 0 in either WKY (*p* = 0.561) or SHR rats (*p* = 0.086). Body weight increased over time in all groups, and the pattern of weight gain did not differ significantly among the groups (Figure 2a).

### 3.2. Changes in Blood Pressure

The linear mixed-effects model revealed a significant main effect of strain (F(1, 35.00) = 226.37, *p* < 0.001), whereas the main effects of treatment (F(1, 35.00) = 2.16, *p* = 0.151) and time (F(1, 35.00) = 2.98, *p* = 0.093) were not significant. The strain × treatment (F(1, 35.00) = 0.60, *p* = 0.443), strain × time (F(1, 35.00) = 3.22, *p* = 0.082), treatment × time (F(1, 35.00) = 0.12, *p* = 0.736), and strain × treatment × time interactions (F(1, 35.00) = 0.08, *p* = 0.777) were not significant. Šídák-adjusted multiple comparisons showed that systolic blood pressure was significantly higher in the SHR(control) group than in the WKY(control) group on both Day 0 (*p* < 0.001) and Day 21 (*p* < 0.001). Similarly, systolic blood pressure was significantly higher in the SHR(BTX) group than in the WKY(BTX) group on both Day 0 (*p* < 0.001) and Day 21 (*p* < 0.001). No significant differences were observed between Day 0 and Day 21 within any of the four groups (all *p* > 0.05) (Figure 2b). 

### 3.3. Tooth-Movement Distance

**Figure 3:**
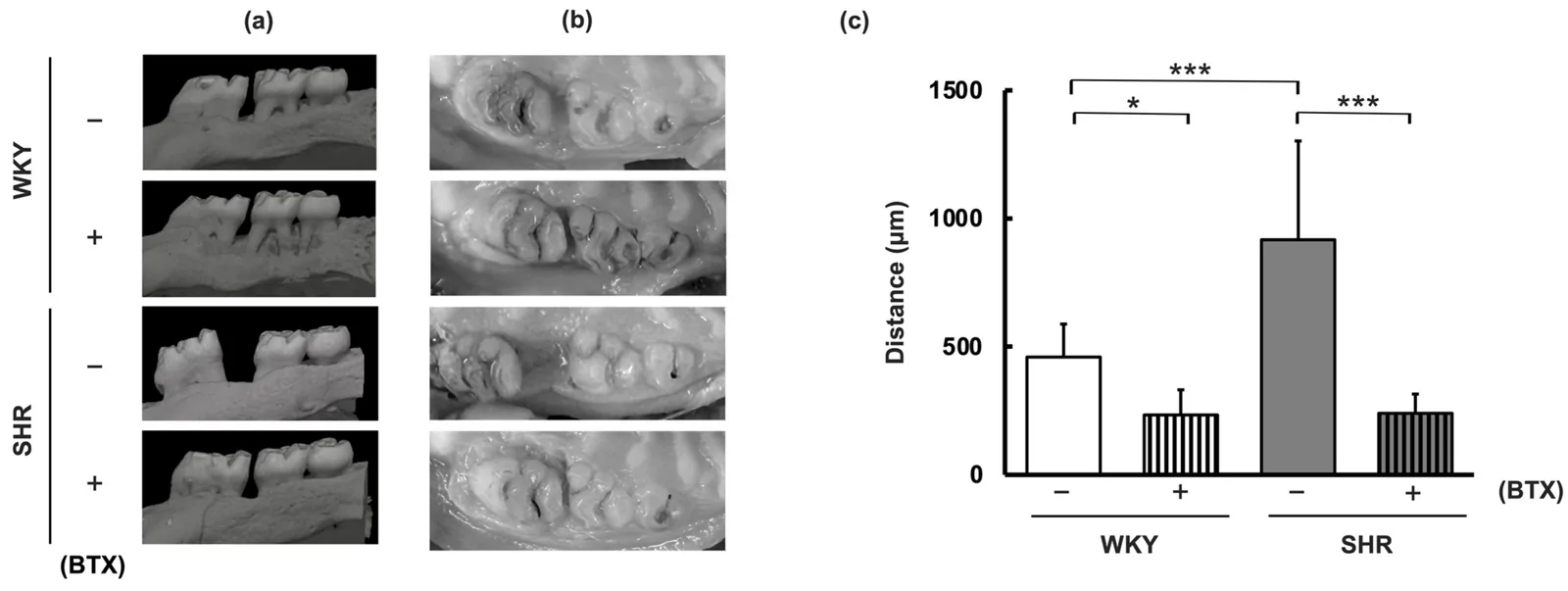
Effects of BTX on the distance of tooth movement. (**a**) μCT images after 21 days of tooth movement. (**b**) Intraoral photographs after 21 days of tooth movement. (**c**) Comparison of the distance of tooth movement after 21 days of tooth movement (WKY(control), *n* = 7; WKY(BTX), *n* = 10; SHR(control), *n* = 12; SHR(BTX), *n* = 10). * *p* < 0.05, *** *p* < 0.001. BTX, butoxamine; SHRs, spontaneously hypertensive rats; WKY, Wistar–Kyoto rats; μCT, micro-computed tomography.

The tooth-movement distances were 0.459 ± 0.129 mm in the WKY(control) group, 0.233 ± 0.098 mm in the WKY(BTX) group, 0.918 ± 0.329 mm in the SHR(control) group, and 0.240 ± 0.075 mm in the SHR(BTX) group. Two-way ANOVA revealed significant main effects of strain (F(1, 35) = 12.44, *p* = 0.001, partial η^2^ = 0.262) and treatment (F(1, 35) = 46.94, *p* < 0.001, partial η^2^ = 0.573). The strain × treatment interaction was significant (F(1, 35) = 11.75, *p* = 0.002, partial η^2^ = 0.251). Šídák-adjusted multiple comparisons showed that tooth movement was significantly greater in the SHR(control) group than in the WKY(control) group (estimated mean difference, 0.459 mm; 95% CI, 0.264–0.654 mm; *p* < 0.001). BTX treatment significantly reduced tooth movement in both WKY rats (WKY(control) vs. WKY(BTX): estimated mean difference, 0.226 mm; 95% CI, 0.024–0.428 mm; *p* = 0.030) and SHR rats (SHR(control) vs. SHR(BTX): estimated mean difference, 0.678 mm; 95% CI, 0.502–0.854 mm; *p* < 0.001) (Figure 3a–c). Because the tooth-movement distance differed among the experimental groups, this difference represents a potential mechanical confounding factor when interpreting subsequent comparisons of OIRR-related outcomes.

### 3.4. Root Length and Root Volume

On the non-loaded side, there were no significant differences in root length or root volume between the WKY and SHR groups (Figure S1). After 21 days of tooth movement, root lengths were 2.256 ± 0.124 mm in the WKY(control) group, 2.233 ± 0.111 mm in the WKY(BTX) group, 2.050 ± 0.097 mm in the SHR(control) group, and 2.173 ± 0.058 mm in the SHR(BTX) group. Two-way ANOVA revealed a significant main effect of strain (F(1, 35) = 17.15, *p* < 0.001, partial η^2^ = 0.329), a non-significant main effect of treatment (F(1, 35) = 2.44, *p* = 0.127, partial η^2^ = 0.065), and a significant strain × treatment interaction (F(1, 35) = 5.18, *p* = 0.029, partial η^2^ = 0.129). Šídák-adjusted multiple comparisons showed that root length was significantly lower in SHR(control) than in WKY(control) (estimated mean difference, 0.205 mm; 95% CI, 0.111–0.300 mm; *p* < 0.001), whereas no significant difference was observed between WKY(control) and WKY(BTX) (estimated mean difference, 0.023 mm; 95% CI, −0.075–0.121 mm; *p* = 0.639). Root length was significantly greater in SHR(BTX) than in SHR(control) (estimated mean difference, 0.123 mm; 95% CI, 0.038–0.208 mm; *p* = 0.006).

**Figure 4:**
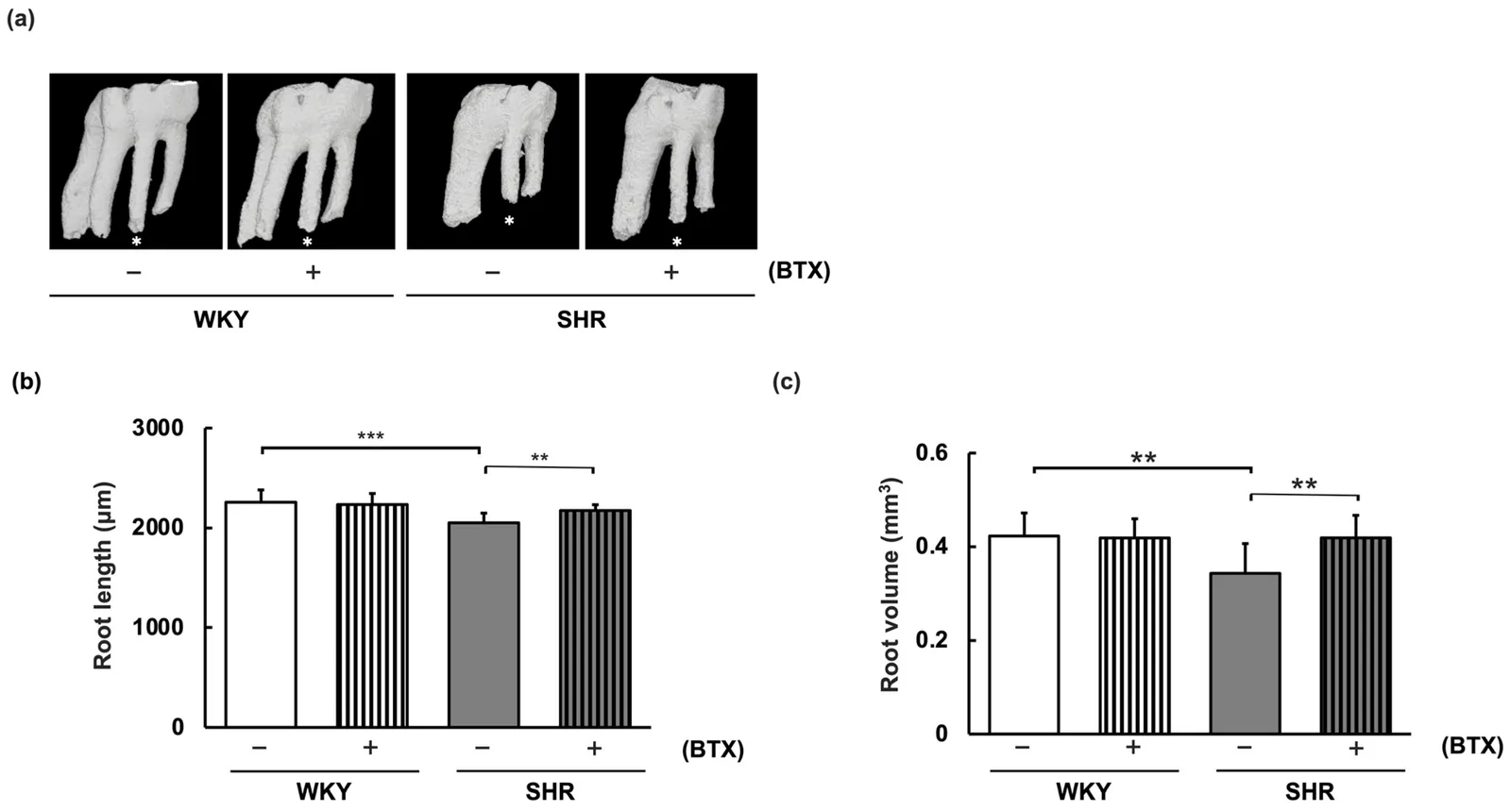
Effects of BTX on root length and root volume. (**a**) μCT images of the maxillary left first molar after 21 days of tooth movement. Asterisks indicate distopalatal roots. (**b**) Comparison of root length after 21 days of tooth movement (WKY(control), *n* = 7; WKY(BTX), *n* = 10; SHR(control), *n* = 12; SHR(BTX), *n* = 10). ** *p* < 0.01, *** *p* < 0.001. (**c**) Comparison of root volume after 21 days of tooth movement (WKY(control), *n* = 7; WKY(BTX), *n* = 10; SHR(control), *n* = 12; SHR(BTX), *n* = 10). ** *p* < 0.01. BTX, butoxamine; SHRs, spontaneously hypertensive rats; WKY, Wistar–Kyoto rats; μCT, micro-computed tomography.

Root volumes were 0.423 ± 0.049 mm^3^ in the WKY(control) group, 0.419 ± 0.041 mm^3^ in the WKY(BTX) group, 0.348 ± 0.061 mm^3^ in the SHR(control) group, and 0.419 ± 0.048 mm^3^ in the SHR(BTX) group. Two-way ANOVA revealed a significant main effect of strain (F(1, 35) = 5.14, *p* = 0.030, partial η^2^ = 0.128), a non-significant main effect of treatment (F(1, 35) = 4.05, *p* = 0.052, partial η^2^ = 0.104), and a significant strain × treatment interaction (F(1, 35) = 5.20, *p* = 0.029, partial η^2^ = 0.129). Šídák-adjusted multiple comparisons showed that root volume was significantly lower in SHR(control) than in WKY(control) (estimated mean difference, 0.076 mm^3^; 95% CI, 0.026–0.125 mm^3^; *p* = 0.004), whereas no significant difference was observed between WKY(control) and WKY(BTX) (estimated mean difference, 0.004 mm^3^; 95% CI, −0.046–0.055 mm^3^; *p* = 0.860). Root volume was significantly greater in SHR(BTX) than in SHR(control) (estimated mean difference, 0.071 mm^3^; 95% CI, 0.027–0.116 mm^3^; *p* = 0.002) (Figure 4b,c).

In the exploratory supplementary comparison in which WKY and SHR rats exhibited comparable tooth-movement distances after different durations of force application, the SHR group showed significantly lower root length and root volume than the WKY group (Figure S2).

### 3.5. Odontoclast Number and Odontoclast Surface

Oc.N/BS values were 9.44 ± 5.08 in the WKY(control) group, 8.55 ± 6.47 in the WKY(BTX) group, 23.47 ± 5.62 in the SHR(control) group, and 6.43 ± 3.81 in the SHR(BTX) group. Two-way ANOVA revealed a significant main effect of strain (F(1, 16) = 6.23, *p* = 0.024, partial η^2^ = 0.280), a significant main effect of treatment (F(1, 16) = 14.13, *p* = 0.002, partial η^2^ = 0.469), and a significant strain × treatment interaction (F(1, 16) = 11.46, *p* = 0.004, partial η^2^ = 0.417). Šídák-adjusted multiple comparisons showed that Oc.N/BS was significantly higher in SHR(control) than in WKY(control) (*p* < 0.001), whereas no significant difference was observed between WKY(control) and WKY(BTX) (*p* = 0.795). Oc.N/BS was significantly lower in SHR(BTX) than in SHR(control) (*p* < 0.001).

**Figure 5:**
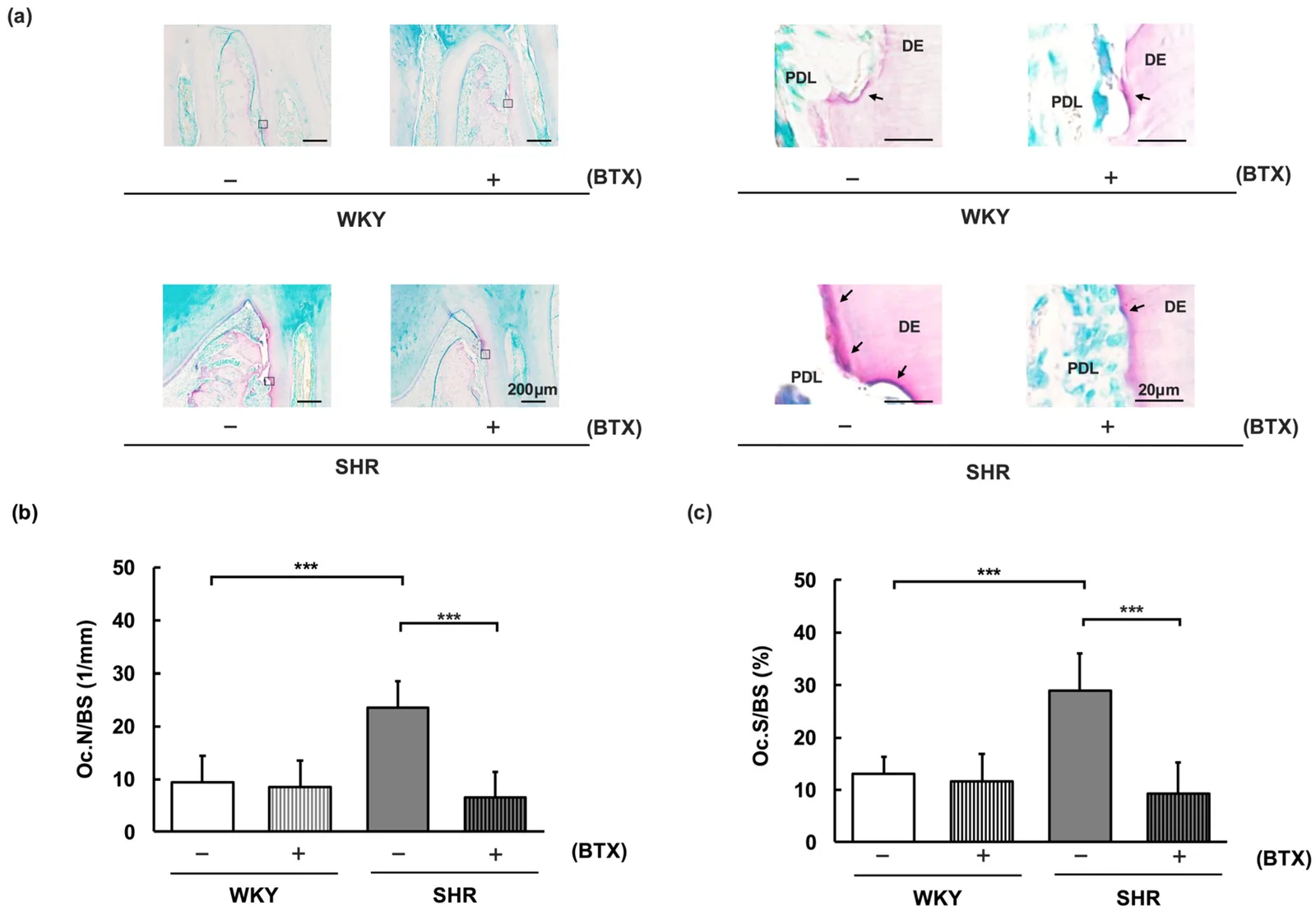
Effects of BTX on odontoclast number and odontoclast surface on the root surface. (**a**) Representative low- and high-magnification TRAP-stained histological sections after 21 days of tooth movement. The boxed area in each low-magnification image indicates the region shown at high magnification. Black arrows indicate TRAP-positive odontoclasts on the root surface. (**b**) Comparison of odontoclast number after 21 days of tooth movement (WKY(control), *n* = 5; WKY(BTX), *n* = 5; SHR(control), *n* = 5; SHR(BTX), *n* = 5). *** *p* < 0.001. (**c**) Comparison of odontoclast surface after 21 days of tooth movement (WKY(control), *n* = 5; WKY(BTX), *n* = 5; SHR(control), *n* = 5; SHR(BTX), *n* = 5). *** *p* < 0.001. BTX, butoxamine; DE, dentin; Oc.N/BS, odontoclasts per root surface; Oc.S/BS, odontoclast surface per root surface; PDL, periodontal ligament; SHRs, spontaneously hypertensive rats; TRAP, tartrate-resistant acid phosphatase; WKY, Wistar–Kyoto rats.

Oc.S/BS values were 13.01 ± 3.31 in the WKY(control) group, 11.69 ± 5.19 in the WKY(BTX) group, 28.87 ± 7.15 in the SHR(control) group, and 9.25 ± 6.02 in the SHR(BTX) group. Two-way ANOVA revealed a significant main effect of strain (F(1, 16) = 7.19, *p* = 0.016, partial η^2^ = 0.310), a significant main effect of treatment (F(1, 16) = 17.53, *p* < 0.001, partial η^2^ = 0.523), and a significant strain × treatment interaction (F(1, 16) = 13.37, *p* = 0.002, partial η^2^ = 0.455). Šídák-adjusted multiple comparisons showed that Oc.S/BS was significantly higher in SHR(control) than in WKY(control) (*p* < 0.001), whereas no significant difference was observed between WKY(control) and WKY(BTX) (*p* = 0.712). Oc.S/BS was significantly lower in SHR(BTX) than in SHR(control) (*p* < 0.001) (Figure 5a–c).

### 3.6. Inflammatory Cytokines

**Figure 6:**
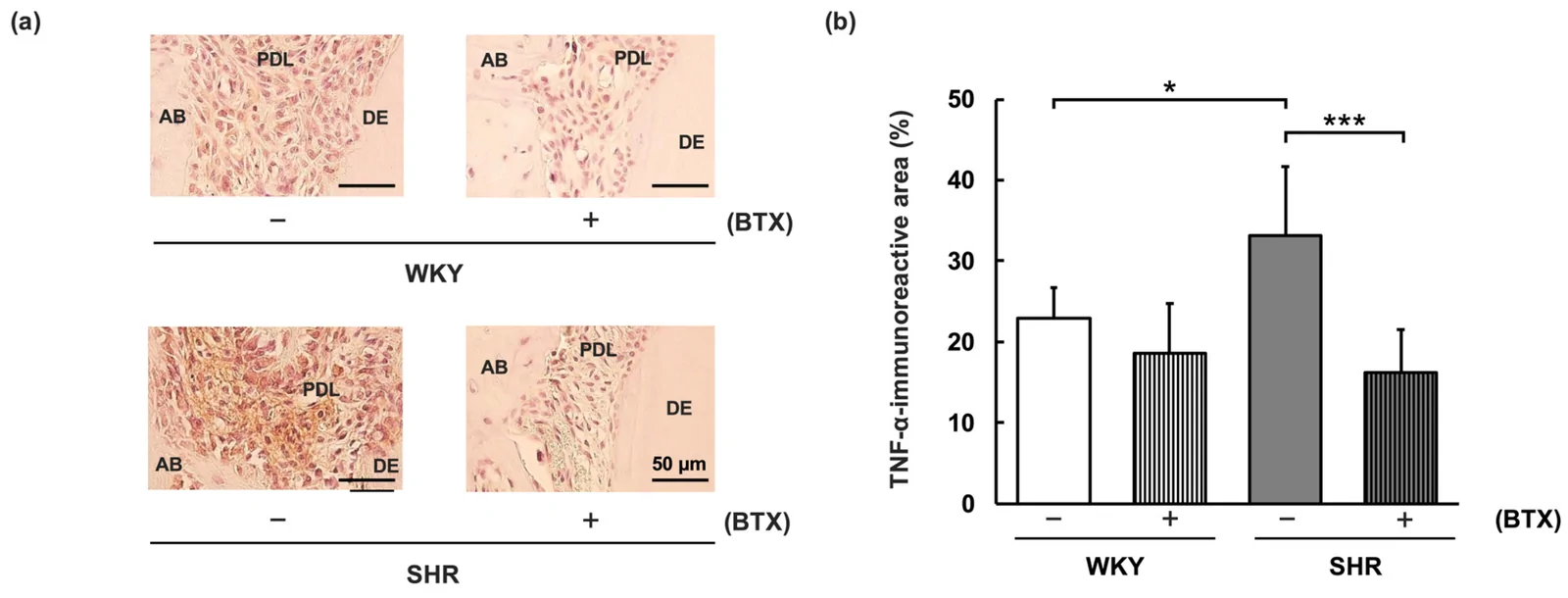
Effects of BTX on TNF-α immunoreactivity in the periodontal ligament. (**a**) Representative TNF-α-immunostained histological sections of the periodontal ligament after 21 days of tooth movement. (**b**) Comparison of the TNF-α-immunoreactive area (%) in the periodontal ligament after 21 days of tooth movement (WKY(control), *n* = 5; WKY(BTX), *n* = 7; SHR(control), *n* = 7; SHR(BTX), *n* = 8). * *p* < 0.05, *** *p* < 0.001. AB, alveolar bone; BTX, butoxamine; DE, dentin; PDL, periodontal ligament; SHRs, spontaneously hypertensive rats; TNF-α, tumor necrosis factor alpha; WKY, Wistar–Kyoto rats.

The TNF-α-positive areas were 22.98 ± 3.75% in the WKY(control) group, 18.51 ± 6.16% in the WKY(BTX) group, 31.79 ± 5.55% in the SHR(control) group, and 16.19 ± 5.29% in the SHR(BTX) group. Two-way ANOVA revealed a non-significant main effect of strain (F(1, 23) = 2.38, *p* = 0.136, partial η^2^ = 0.094), a significant main effect of treatment (F(1, 23) = 22.80, *p* < 0.001, partial η^2^ = 0.498), and a significant strain × treatment interaction (F(1, 23) = 7.03, *p* = 0.014, partial η^2^ = 0.234). Šídák-adjusted multiple comparisons showed that the TNF-α-positive area was significantly greater in SHR(control) than in WKY(control) (*p* = 0.010), whereas no significant difference was observed between WKY(control) and WKY(BTX) (*p* = 0.170). The TNF-α-positive area was significantly smaller in SHR(BTX) than in SHR(control) (*p* < 0.001) (Figure 6a,b).

**Figure 7:**
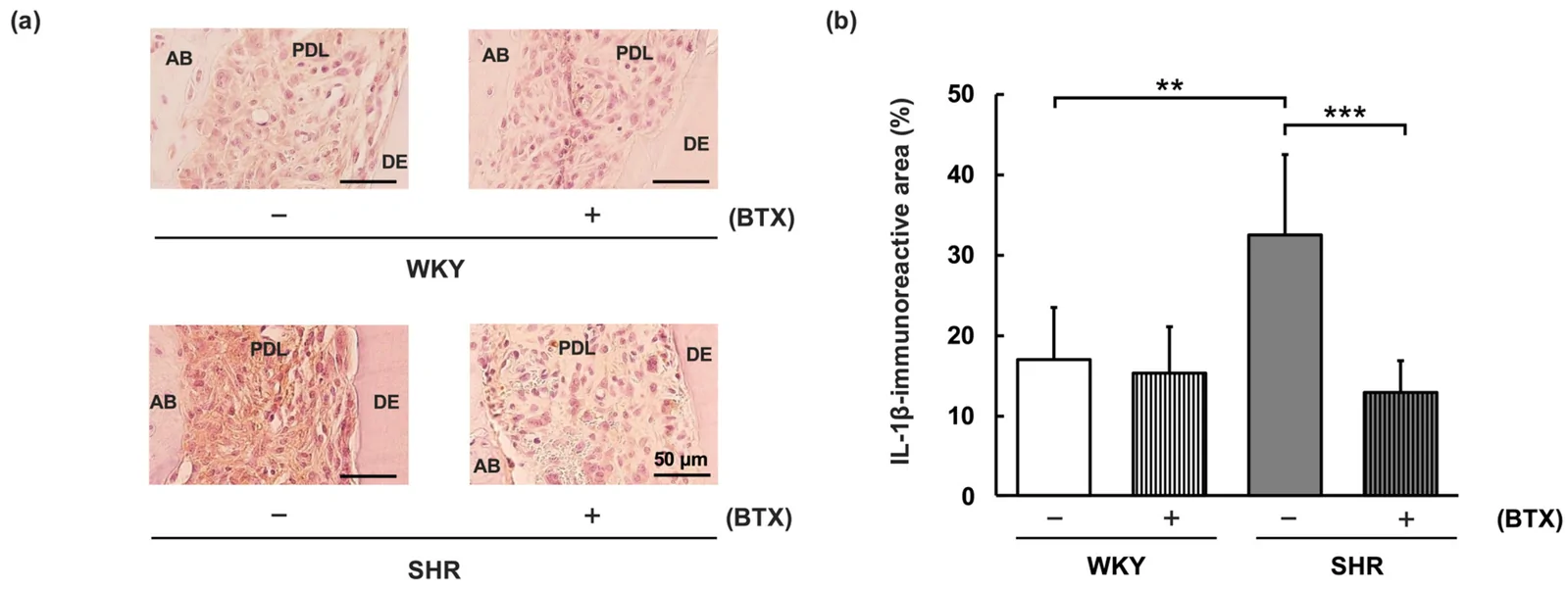
Effects of BTX on IL-1β immunoreactivity in the periodontal ligament. (**a**) Representative IL-1β-immunostained histological sections of the periodontal ligament after 21 days of tooth movement. (**b**) Comparison of the IL-1β-immunoreactive area (%) in the periodontal ligament after 21 days of tooth movement (WKY(control), *n* = 5; WKY(BTX), *n* = 6; SHR(control), *n* = 6; SHR(BTX), *n* = 7). ** *p* < 0.01, *** *p* < 0.001. AB, alveolar bone; BTX, butoxamine; DE, dentin; IL-1β, interleukin-1 beta; PDL, periodontal ligament; SHRs, spontaneously hypertensive rats; WKY, Wistar–Kyoto rats.

The IL-1β-positive areas were 17.00 ± 6.51% in the WKY(control) group, 15.32 ± 5.77% in the WKY(BTX) group, 32.42 ± 10.02% in the SHR(control) group, and 12.82 ± 3.98% in the SHR(BTX) group. Two-way ANOVA revealed a significant main effect of strain (F(1, 20) = 5.30, *p* = 0.032, partial η^2^ = 0.209), a significant main effect of treatment (F(1, 20) = 14.35, *p* = 0.001, partial η^2^ = 0.418), and a significant strain × treatment interaction (F(1, 20) = 10.18, *p* = 0.005, partial η^2^ = 0.337). Šídák-adjusted multiple comparisons showed that the IL-1β-positive area was significantly greater in SHR(control) than in WKY(control) (*p* = 0.001), whereas no significant difference was observed between WKY(control) and WKY(BTX) (*p* = 0.690). The IL-1β-positive area was significantly smaller in SHR(BTX) than in SHR(control) (*p* < 0.001) (Figure 7a,b).

### 3.7. Cementocyte Density and Empty Lacunae Ratio

Cementocyte densities were 0.00987 ± 0.00076 cells/μm^2^ in the WKY(control) group, 0.00858 ± 0.00177 cells/μm^2^ in the WKY(BTX) group, 0.00691 ± 0.00198 cells/μm^2^ in the SHR(control) group, and 0.00971 ± 0.00118 cells/μm^2^ in the SHR(BTX) group. Two-way ANOVA revealed a non-significant main effect of strain (F(1, 17) = 1.99, *p* = 0.176, partial η^2^ = 0.105), a non-significant main effect of treatment (F(1, 17) = 1.35, *p* = 0.261, partial η^2^ = 0.074), and a significant strain × treatment interaction (F(1, 17) = 9.89, *p* = 0.006, partial η^2^ = 0.368). Šídák-adjusted multiple comparisons showed that the cementocyte density was significantly lower in SHR(control) than in WKY(control) (*p* = 0.006), whereas no significant difference was observed between WKY(control) and WKY(BTX) (*p* = 0.188). The cementocyte density was significantly greater in SHR(BTX) than in SHR(control) (*p* = 0.006).

**Figure 8:**
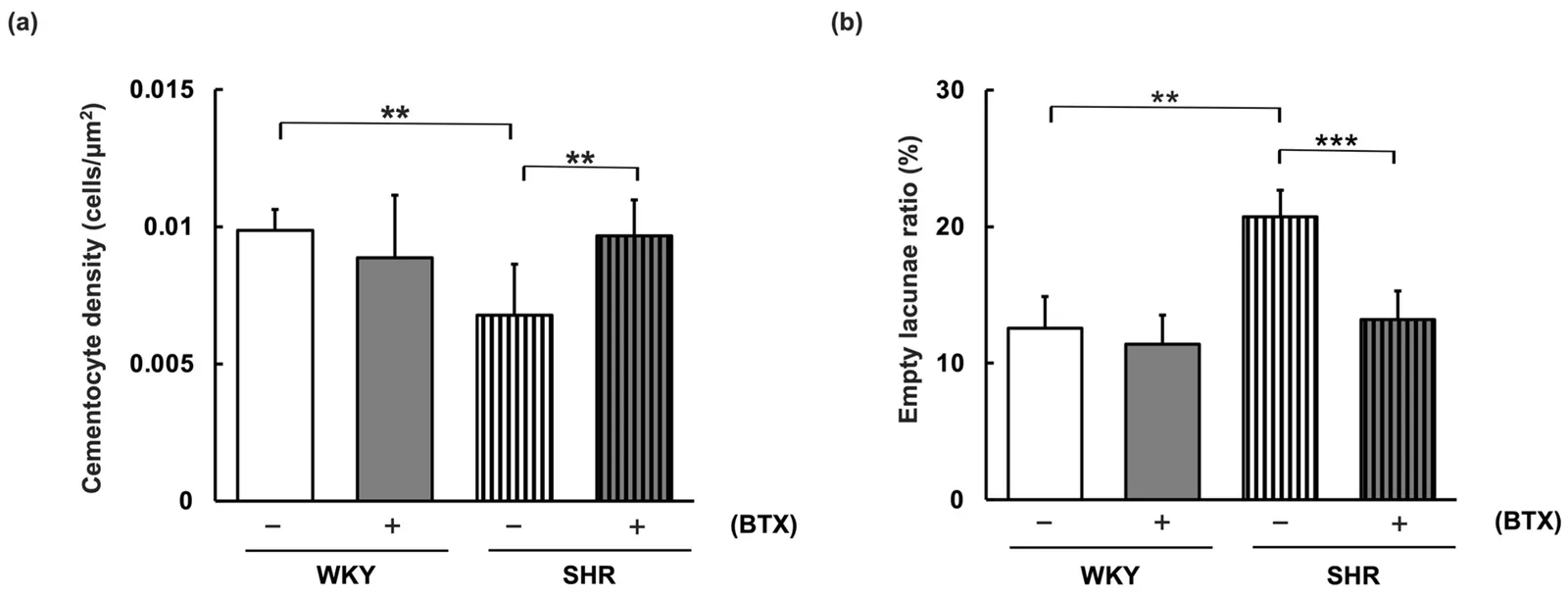
Effects of BTX on cementocyte density and the empty lacunae ratio in cellular cementum. (**a**) Comparison of cementocyte density after 21 days of tooth movement (WKY(control), *n* = 5; WKY(BTX), *n* = 5; SHR(control), *n* = 5; SHR(BTX), *n* = 6). ** *p* < 0.01. (**b**) Comparison of the empty lacunae ratio after 21 days of tooth movement (WKY(control), *n* = 5; WKY(BTX), *n* = 5; SHR(control), *n* = 5; SHR(BTX), *n* = 6). ** *p* < 0.01, *** *p* < 0.001. BTX, butoxamine; SHRs, spontaneously hypertensive rats; WKY, Wistar–Kyoto rats.

The empty lacunae ratios were 14.22 ± 4.22% in the WKY(control) group, 10.90 ± 2.14% in the WKY(BTX) group, 20.28 ± 2.90% in the SHR(control) group, and 12.30 ± 2.89% in the SHR(BTX) group. Two-way ANOVA revealed a significant main effect of strain (F(1, 17) = 7.48, *p* = 0.014, partial η^2^ = 0.306), a significant main effect of treatment (F(1, 17) = 17.17, *p* < 0.001, partial η^2^ = 0.502), and a non-significant strain × treatment interaction (F(1, 17) = 2.93, *p* = 0.105, partial η^2^ = 0.147). Šídák-adjusted multiple comparisons showed that the empty lacunae ratio was significantly higher in SHR(control) than in WKY(control) (*p* = 0.007), whereas no significant difference was observed between WKY(control) and WKY(BTX) (*p* = 0.111). The empty lacunae ratio was significantly lower in SHR(BTX) than in SHR(control) (*p* < 0.001) (Figure 8a,b).

### 3.8. TUNEL-Positive Cells

**Figure 9:**
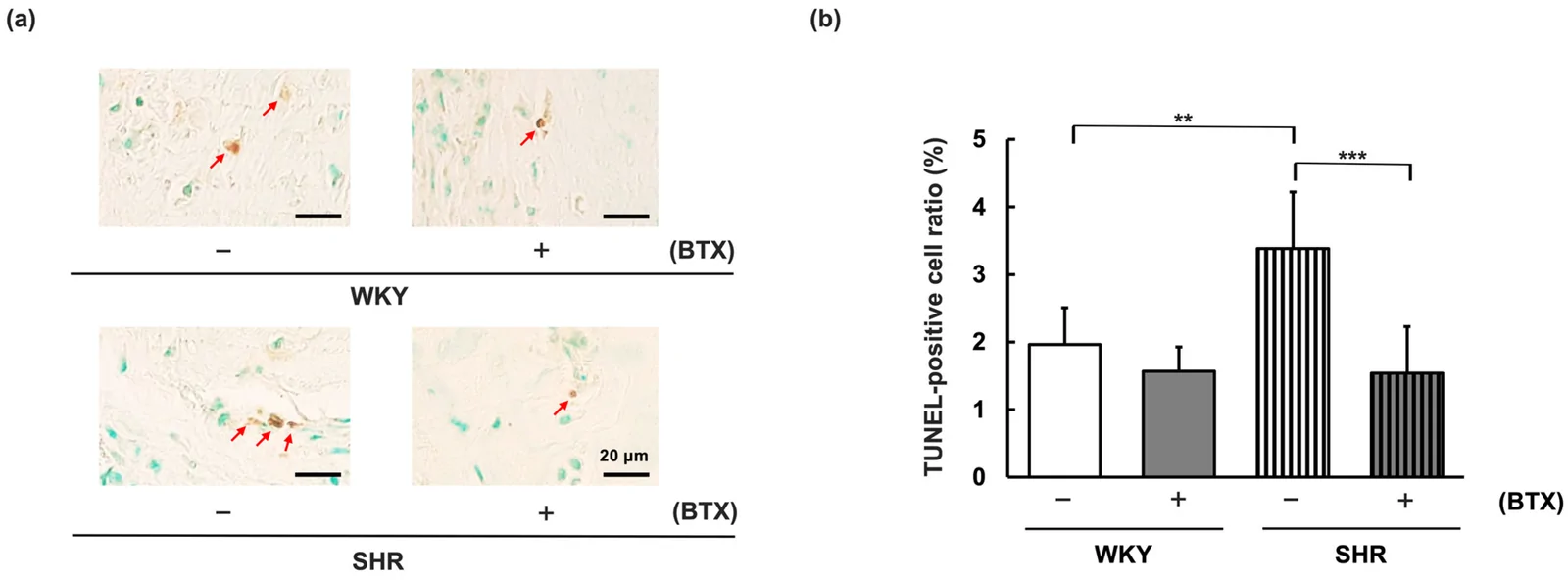
Effects of BTX on TUNEL-positive cells in cellular cementum. (**a**) TUNEL-stained histological sections of cellular cementum after 21 days of tooth movement. Red arrows indicate TUNEL-positive cells located within cementocyte lacunae in the cellular cementum. (**b**) Comparison of the TUNEL-positive cell ratio (%) after 21 days of tooth movement (WKY(control), *n* = 5; WKY(BTX), *n* = 6; SHR(control), *n* = 6; SHR(BTX), *n* = 6). ** *p* < 0.01, *** *p* < 0.001. BTX, butoxamine; SHRs, spontaneously hypertensive rats; TUNEL, terminal deoxynucleotidyl transferase dUTP nick end labeling; WKY, Wistar–Kyoto rats.

The TUNEL-positive cell ratios were 1.964 ± 0.544% in the WKY(control) group, 1.568 ± 0.361% in the WKY(BTX) group, 3.383 ± 0.837% in the SHR(control) group, and 1.538 ± 0.690% in the SHR(BTX) group. Two-way ANOVA revealed a significant main effect of strain (F(1, 19) = 6.83, *p* = 0.017, partial η^2^ = 0.264), a significant main effect of treatment (F(1, 19) = 17.67, *p* < 0.001, partial η^2^ = 0.482), and a significant strain × treatment interaction (F(1, 19) = 7.39, *p* = 0.014, partial η^2^ = 0.280). Šídák-adjusted multiple comparisons showed that the TUNEL-positive cell ratio was significantly higher in SHR(control) than in WKY(control) (*p* = 0.002), whereas no significant difference was observed between WKY(control) and WKY(BTX) (*p* = 0.318). The TUNEL-positive cell ratio was significantly lower in SHR(BTX) than in SHR(control) (*p* < 0.001) (Figure 9a,b).

### 3.9. β2-AR Immunoreactivity

The β2-AR-positive areas were 28.87 ± 3.32% in the WKY(control) group, 15.60 ± 3.40% in the WKY(BTX) group, 30.23 ± 5.86% in the SHR(control) group, and 17.07 ± 3.71% in the SHR(BTX) group. Two-way ANOVA revealed a non-significant main effect of strain (F(1, 20) = 0.68, *p* = 0.419, partial η^2^ = 0.033), a significant main effect of treatment (F(1, 20) = 59.38, *p* < 0.001, partial η^2^ = 0.748), and a non-significant strain × treatment interaction (F(1, 20) = 0.001, *p* = 0.977, partial η^2^ < 0.001). Šídák-adjusted multiple comparisons showed no significant difference in the β2-AR-positive area between WKY(control) and SHR(control) (*p* = 0.579). BTX treatment significantly reduced the β2-AR-positive area in both WKY rats (WKY(control) vs. WKY(BTX), *p* < 0.001) and SHR rats (SHR(control) vs. SHR(BTX), *p* < 0.001) (Figure S3).

## 4. Discussion

The involvement of the sympathetic nervous system in bone metabolism and orthodontic tooth movement has recently attracted growing attention. Sato et al. reported that the distance of tooth movement was increased in SHRs and that the administration of BTX, a selective β2-AR antagonist, suppressed both tooth movement and alveolar bone resorption [8]. Kondo et al. also demonstrated that TH-positive nerve fibers in the periodontal ligament were increased during tooth movement and that sympathetic nervous signaling contributed to osteoclast activation and enhanced tooth movement [9]. In the present study, we used the experimental tooth-movement model described by Dunn et al. [18] and Sato et al. [8] to investigate OIRR in the SHR model and the effects of β2-AR blockade. The results showed that SHR exhibited a greater distance of tooth movement and reduced root length and root volume compared with WKY rats. On the other hand, the increases in the tooth-movement distance and the changes in root morphology observed in SHRs were attenuated following BTX administration. These findings suggest that the SHR phenotype is associated with enhanced orthodontic tooth movement and greater root morphological changes consistent with OIRR, while the attenuation observed following BTX administration supports a potential contribution of β2-adrenergic signaling to these responses. In our previous study, TH-positive nerve fibers in the periodontal ligament were more prominent in SHRs than in WKY rats during orthodontic tooth movement, consistent with altered sympathetic/adrenergic signaling in the SHR model [8]. In contrast, β2-AR immunoreactivity in the periodontal ligament was comparable between the WKY(control) and SHR(control) groups in the present study, whereas BTX reduced β2-AR immunoreactivity in both strains (Figure S3). These findings suggest that the greater OIRR observed in SHRs may be associated with altered adrenergic signaling involving β2-ARs rather than with greater β2-AR immunoreactivity itself.

Root resorption is known to occur as a result of sterile inflammation in the periodontal ligament on the compression side [12,19]. Zhong et al. have described that orthodontic force increases the compressive hydrostatic pressure within the periodontal ligament and that root resorption becomes more likely to occur when this pressure exceeds the capillary pressure [14]. In the present study, SHRs showed reductions in root length and root volume, suggesting that the SHR phenotype may be associated with greater susceptibility to root morphological changes related to OIRR during orthodontic tooth movement. In addition, the attenuation of the reductions in root length and root volume following BTX administration supports a potential contribution of β2-AR signaling to these root morphological changes. However, because BTX also reduced tooth movement, the lower degree of OIRR-related change in BTX-treated animals may reflect differences in local mechanical exposure, as well as biological effects of β2-AR blockade. Although the exploratory comparison in Figure S2 suggests that the greater root morphological changes in SHRs were not entirely explained by final tooth displacement, the different durations of force application preclude the complete separation of mechanical and adrenergic effects. Furthermore, the small sample size of this supplementary comparison limits the strength of its interpretation. A further limitation is that force decay was not quantitatively measured during the 21-day period, although appliance integrity was monitored throughout the experiment; therefore, temporal changes in the actual orthodontic force delivered to the teeth cannot be excluded. Furthermore, although the same nominal force of 50 gf was applied to both strains, identical force does not necessarily produce equivalent periodontal stress if anatomical or biomechanical differences exist between WKY and SHR rats. More generally, clinical studies of surgically–orthodontically treated impacted teeth have also emphasized the relevance of local anatomical and treatment-related factors to periodontal outcomes [20]. In addition, root length and total root volume are indirect morphological indicators of OIRR and do not directly quantify resorption lacunae or the resorbed root surface or volume; therefore, the μCT findings should be interpreted as morphological evidence supportive of OIRR. Although the same segmentation threshold was applied to all specimens and μCT measurements were performed blinded to group allocation, the formal intra- or inter-examiner reproducibility of these measurements was not assessed.

We then evaluated changes in odontoclasts on the root surface on the compression side. During the early stage of tooth movement, periodontal ligament compression causes impaired blood flow and hyalinization, thereby contributing to inflammatory cell infiltration and odontoclast induction [12,19]. Regarding the relationship between the sympathetic nervous system and bone metabolism, Togari has reported that β2-ARs are expressed on osteoblasts and osteoclasts and that sympathetic neurotransmitters such as noradrenaline may promote osteoclastogenesis and bone-resorptive activity through β2-AR signaling [21]. In the present study, SHRs showed increases in the odontoclast number and odontoclast surface, and these increases were attenuated following BTX administration. Therefore, the increased odontoclast number and surface observed in SHRs, together with their attenuation following BTX administration, support a potential contribution of β2-adrenergic signaling to odontoclast-related root resorptive responses in this model. It should be noted that the odontoclast parameters evaluated in this study reflect root-resorptive activity and are distinct from osteoclast-mediated alveolar bone remodeling, which more directly contributes to orthodontic tooth movement. Therefore, the absence of significant changes in the odontoclast number and surface in WKY rats after BTX treatment does not necessarily conflict with the observed reduction in the tooth-movement distance.

Inflammatory cytokines are also considered to play an important role in the progression of root resorption. Rogers et al. found, in an LPS-induced periodontitis model, increased expression of TNF-α, interleukin (IL)-1β, and IL-6 and an increase in TRAP-positive cells, accompanied by enhanced bone resorption [22]. Takeguchi et al. also reported that, in an experimental periodontitis model using SHRs, BTX administration reduced the expression of TNF-α, IL-1β, and IL-6, decreased the number of osteoclasts, and suppressed alveolar bone resorption [11]. Furthermore, Muramatsu et al. demonstrated that the administration of guanabenz, which reduces sympathetic nervous activity, decreased the expression of TNF-α, IL-1β, and IL-6, decreased the number of TH-positive cells, and suppressed alveolar bone resorption in an experimental periodontitis model using SHRs [23]. In the present study, SHRs likewise showed greater TNF-α- and IL-1β-positive areas in the periodontal ligament, and these positive areas were smaller following BTX administration. These findings support a potential contribution of β2-adrenergic signaling to the periodontal inflammatory response associated with OIRR; however, the present data do not establish a direct causal pathway linking β2-adrenergic signaling, cytokine responses, and odontoclast induction. Immunohistochemistry reflects tissue immunoreactivity rather than direct cytokine secretion, and the absence of cell-type-specific markers such as CD68 precluded an identification of the cellular sources of TNF-α and IL-1β. Therefore, the lower TNF-α and IL-1β immunoreactivity observed following BTX administration should be interpreted as a difference in tissue immunoreactivity rather than as direct evidence of altered cytokine production or secretion; approaches such as in situ hybridization and cell-type-specific localization analyses may provide complementary information.

In the present study, we also evaluated the cementocyte density, the empty lacunae ratio, and the number of TUNEL-positive cells in the cellular cementum. On the compression side under orthodontic force, impaired blood flow and hyalinization are known to occur and induce cell death [19,24]. Osteocytes respond to mechanical stimuli and changes in the local environment, and the apoptotic death of these cells has been suggested to be involved in tissue remodeling [25]. Hamaya et al. noted the presence of TUNEL-positive cells and apoptotic changes in alveolar bone osteocytes adjacent to the hyalinized periodontal ligament during tooth movement [26]. Moin et al. also found an increased number of TUNEL-positive osteocytes in a tooth-movement model, followed by an increase in TRAP-positive osteoclast formation, suggesting the possible involvement of osteocyte death in the induction of bone resorption [27].

Cementocytes share morphological characteristics with osteocytes and may respond to changes in the mechanical environment [28]. Matsuzawa et al. found that the numbers of cleaved caspase-3-positive and ssDNA-positive cementocytes increased in the cellular cementum on the compression side during tooth movement, followed by root resorption mediated by TRAP-positive cells [24]. An increase in empty lacunae is also thought to reflect cementocyte loss and impaired maintenance of cementum homeostasis [24,28,29]. More recently, Wang et al. demonstrated that force-loaded cementocytes can promote osteoclastogenic responses through a SphK1/mitophagy-dependent mechanism, further supporting an active role of cementocytes in OIRR [30]. However, the apparent frequency of empty lacunae may also be influenced by the histological processing and section orientation; therefore, an increased empty lacunae ratio should be interpreted cautiously as an indicator consistent with cementocyte loss or damage rather than as direct evidence of cell loss. In the present study, SHRs showed a decreased cementocyte density, an increased empty lacunae ratio, and an increased number of TUNEL-positive cells in the cellular cementum. The TUNEL-positive cells evaluated in the present study were located within cementocyte lacunae in the cellular cementum and were not interpreted as osteoclasts or odontoclasts. Because TUNEL detects DNA fragmentation and is not specific for apoptosis, the increased number of TUNEL-positive cells within cementocyte lacunae should be interpreted as evidence of cellular DNA fragmentation or cell death rather than definitive evidence of cementocyte apoptosis. However, these changes were attenuated following BTX administration. Thus, the concurrent changes in the cementocyte density, empty lacunae ratio, and TUNEL positivity support an association between cementocyte damage and the enhanced OIRR phenotype in SHRs, with attenuation following BTX administration suggesting a potential contribution of β2-adrenergic signaling; however, the present data do not establish that cementocyte damage causally precedes odontoclast-mediated root resorption.

Taken together, the greater OIRR-related changes observed in SHRs were accompanied by greater TNF-α and IL-1β immunoreactivity in the periodontal ligament, increased odontoclasts on the root surface, decreased cementocyte density, and an increased TUNEL-positive cell ratio in the cellular cementum. These findings indicate that the greater OIRR phenotype observed in SHRs was accompanied by inflammatory, odontoclast-related, and cementocyte-related changes. The attenuation of these changes following BTX administration supports a potential contribution of β2-adrenergic signaling to this response.

The novelty of this study lies in demonstrating greater OIRR-related changes in SHRs than in WKY rats and showing that these changes were attenuated by β2-AR blockade, thereby supporting a potential role of β2-adrenergic signaling. Given the increasing number of adult orthodontic patients, the present findings suggest that the systemic adrenergic status may warrant further investigation as a potential modifier of susceptibility to orthodontic root resorption. However, some limitations should be kept in mind. Because this study used only young male rats and a 21-day experimental tooth-movement model, the findings may not be generalizable to female rats or other age groups, and caution is required when extrapolating them to humans. Human hypertension is heterogeneous and is frequently influenced by antihypertensive treatment, age, and metabolic comorbidities, which are not represented in the present model. Because BTX was administered systemically, effects outside the periodontal tissues cannot be excluded. Notably, BTX did not normalize the elevated systolic blood pressure in SHRs, suggesting that the attenuation of OIRR-related changes cannot be explained solely by the normalization of systemic hypertension. Moreover, blood pressure was measured only at the beginning and end of the tooth-movement period; therefore, transient or intermediate cardiovascular changes during daily BTX administration could not be assessed. Although β2-AR immunoreactivity was detected in the periodontal ligament, the present study did not determine its cell-type-specific localization, including whether the observed β2-AR immunoreactivity was localized to cementocytes. Importantly, reduced β2-adrenergic responsiveness in hypertension does not necessarily indicate reduced receptor expression, as impaired β-adrenergic responsiveness has been reported without a corresponding reduction in β-adrenoceptor density [31]. Therefore, it remains unclear whether β2-adrenergic signaling directly affects cementocytes or influences them indirectly through inflammatory or other local mediators. The present findings should therefore be interpreted as demonstrating associations among the SHR phenotype, β2-adrenergic blockade, inflammatory and odontoclast-related changes, and OIRR, rather than establishing a definitive mechanistic pathway. In particular, the immunohistochemical findings provide indirect evidence and do not establish cell-type-specific signaling or direct causal relationships among β2-AR signaling, inflammatory cytokines, cementocyte-related changes, and odontoclast-mediated root resorption. Another limitation is that sympathetic nervous activity itself was not directly measured in the present animals. Further studies combining a direct assessment of sympathetic activity with the cell-type-specific localization of β2-AR and an analysis of downstream signaling will be necessary to clarify the mechanisms linking adrenergic signaling to cementocyte-related changes and OIRR.

Taken together, these findings suggest that the SHR phenotype is associated with greater OIRR-related changes under orthodontic force and that β2-adrenergic signaling may contribute to these responses. These results support further investigation of adrenergic signaling as a potential biological modifier of orthodontic root resorption.

## 5. Conclusions

This study demonstrated greater OIRR-related root morphological changes in SHRs than in WKY rats during experimental orthodontic tooth movement. SHRs showed a reduced root length and root volume, an increased odontoclast number and surface, greater TNF-α and IL-1β immunoreactivity, a decreased cementocyte density, an increased empty lacunae ratio, and more TUNEL-positive cells within cementocyte lacunae. These changes were attenuated following the administration of butoxamine, a β2-AR antagonist.

These findings suggest that the SHR phenotype is associated with greater OIRR-related changes and that β2-adrenergic signaling may contribute to these responses. Further studies are warranted to determine whether adrenergic signaling modifies susceptibility to orthodontic root resorption.

## Data Availability

The data underlying the findings of this study are available from the corresponding author upon reasonable request.

## Supplementary Materials

The following supporting information can be downloaded at https://www.mdpi.com/article/10.3390/jcm15187271/s1, Figure S1: Comparison of root length and root volume on the non-loaded side; Figure S2: Exploratory comparison of root length and root volume between WKY and SHR rats showing comparable tooth-movement distances after different durations of orthodontic force application; Figure S3: β2-AR immunoreactivity and β2-AR-positive area (%) in the periodontal ligament of WKY and SHR rats with or without BTX administration.

## Abbreviations

The following abbreviations are used in this manuscript:

3D	Three-dimensional
BTX	Butoxamine
IL	Interleukin
OIRR	Orthodontically induced inflammatory root resorption
Oc.N/BS	Odontoclasts per root surface
Oc.S/BS	Odontoclast surface per root surface
ROI	Region of interest
SHR	Spontaneously hypertensive rats
TNF	Tumor necrosis factor
TRAP	Tartrate-resistant acid phosphatase
TUNEL	Terminal deoxynucleotidyl transferase dUTP nick end labeling
WKY	Wistar–Kyoto
β_2_-AR	β_2_-Adrenergic receptor
μCT	Micro-computed tomography
