# Supplementary material for: Orthodontic Root Resorption in Spontaneously Hypertensive Rats: Modulation by β2-Adrenergic Receptor Blockade
Source: J Clin Med. 2026 Sep 18;15(18):7271. doi: 10.3390/jcm15187271 (PMC13607296; doi:10.3390/jcm15187271)
Supplement: Supplementary file 1 [file jcm-15-07271-s001.zip › jcm-4509439-supplementary.pdf]

Supplementary Materials

Orthodontic Root Resorption in Spontaneously Hypertensive Rats: Modulation by β2-Adrenergic Receptor Blockade

Tomoma Yoshida, Takuma Sato, Mifumi Takahashi, Shota Ichikawa, Masako Tabuchi and Ken Miyazawa

Contents

Figures S1, S2 and S3

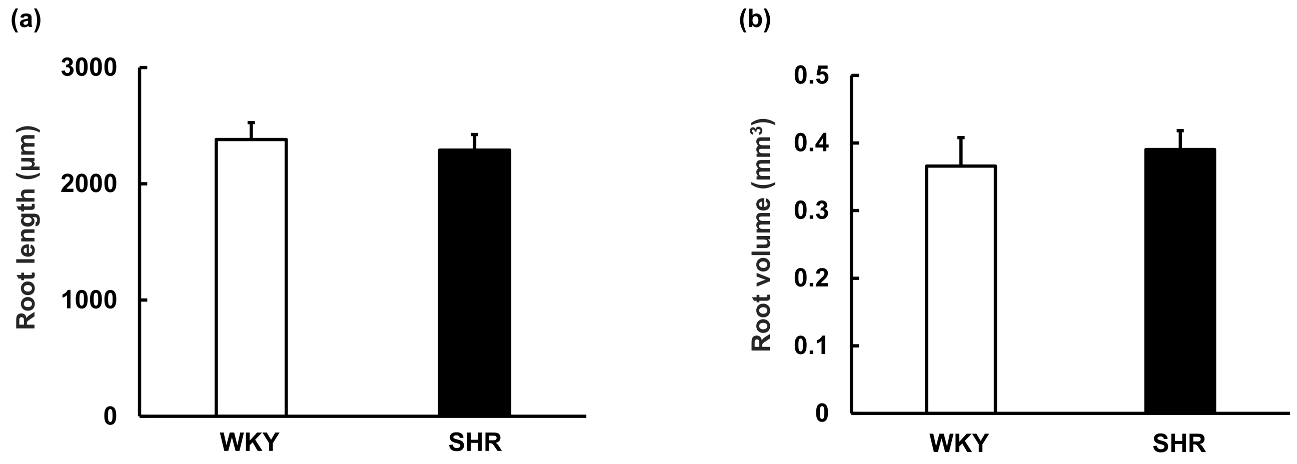

Figure S1. Comparison of root length and root volume on the non-loaded side. (**a**) Comparison of root length on the non-loaded side (WKY, n = 7; SHR, n = 9). (**b**) Comparison of root volume on the non-loaded side (WKY, n = 7; SHR, n = 9). SHR, spontaneously hypertensive rats; WKY, Wistar-Kyoto rats.

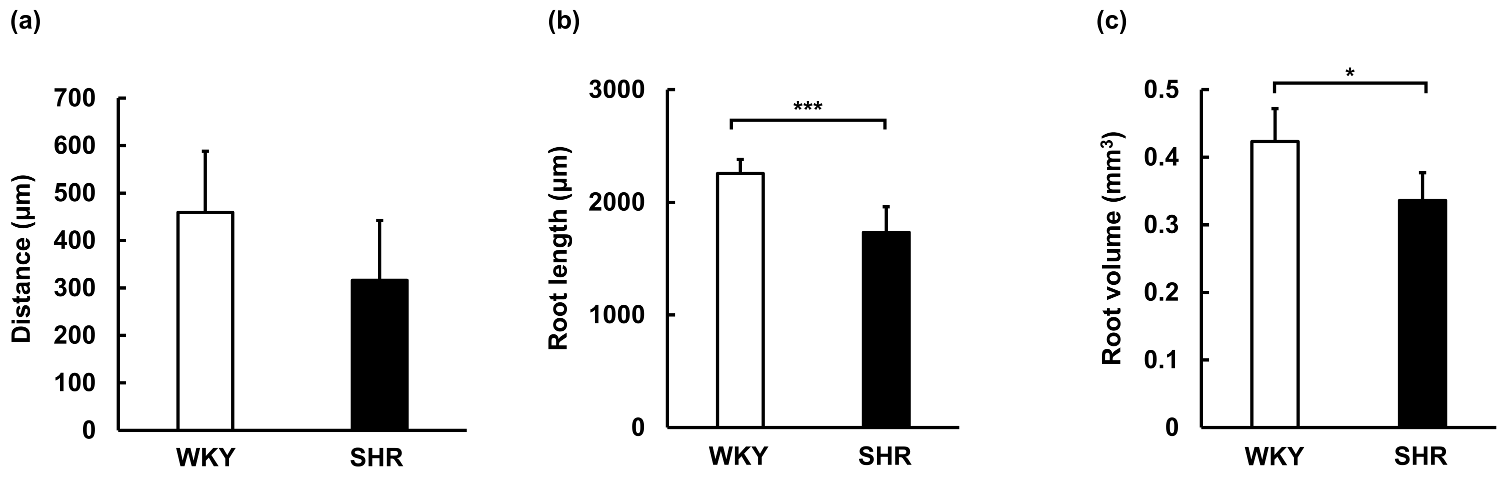

Figure S2. Exploratory comparison of root length and root volume between WKY and SHR groups under conditions in which a 50-gf orthodontic force was applied and the distance of tooth movement was comparable. (**a**) Comparison of the distance of tooth movement at the end of tooth movement (WKY: 21 days of force application, SHR: 13 days of force application; WKY: n = 7, SHR: n = 4). (**b**) Comparison of root length at the end of tooth movement (WKY, n = 7; SHR, n = 4). ***$p < 0.001$. (**c**) Comparison of root volume at the end of tooth movement (WKY, n = 7; SHR, n = 4). *$p < 0.05$. SHR, spontaneously hypertensive rats; WKY, Wistar-Kyoto rats.

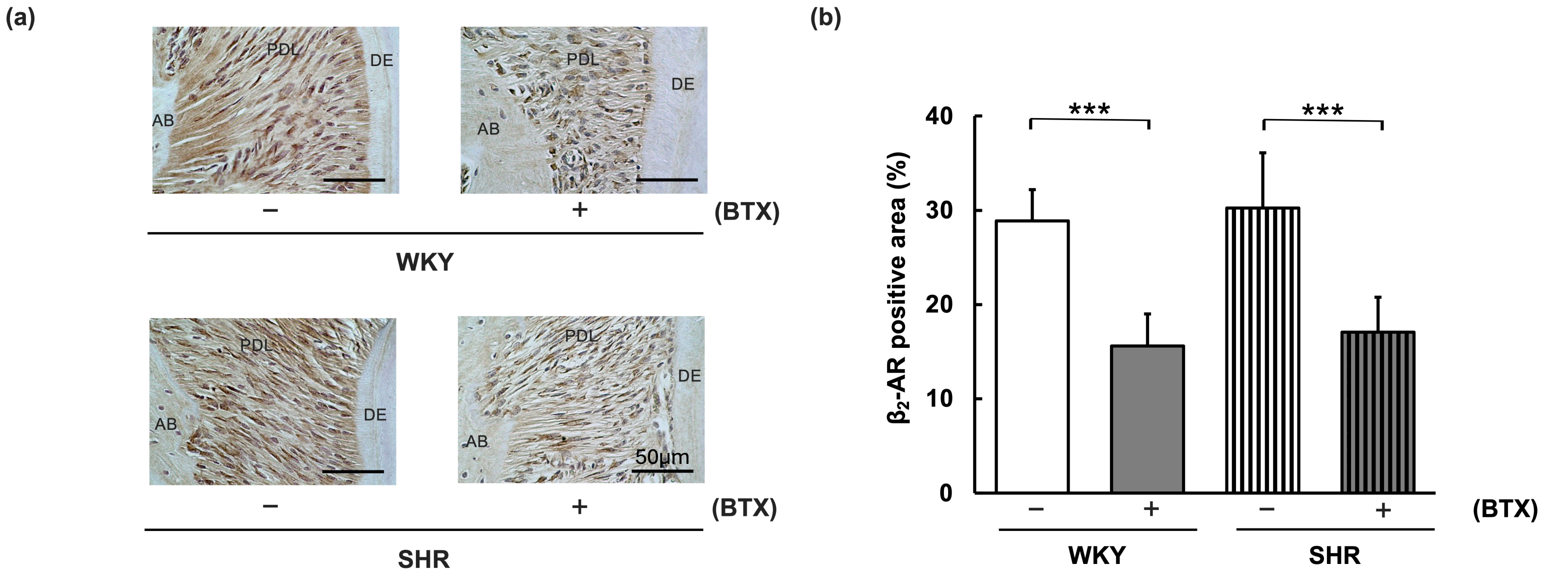

Figure S3. Effects of BTX on β2-AR immunoreactivity in the periodontal ligament. (a) Representative β2-AR-immunostained histological sections of the periodontal ligament after 21 days of tooth movement. (b) Comparison of the β2-AR-positive area (%) in the periodontal ligament after 21 days of tooth movement (WKY(control), n = 6; WKY(BTX), n = 6; SHR(control), n = 6; SHR(BTX), n = 6). ***$p < 0.001$. β2-AR, β2-adrenergic receptor; BTX, butoxamine; PDL, periodontal ligament; SHR, spontaneously hypertensive rats; WKY, Wistar-Kyoto rats.
